# Diurnal temperature range and childhood asthma: a time-series study

**DOI:** 10.1186/1476-069X-12-12

**Published:** 2013-02-01

**Authors:** Zhiwei Xu, Cunrui Huang, Hong Su, Lyle R Turner, Zhen Qiao, Shilu Tong

**Affiliations:** 1 School of Public Health and Social Work& Institute of Health and Biomedical Innovation, Queensland University of Technology, Kelvin Grove, Brisbane, Australia; 2 Centre for Environment and Population Health, School of Environment, Griffith University, Brisbane, Australia; 3 Department of Health Statistics and Epidemiology, School of Public Health, Anhui Medical University, Hefei, China

**Keywords:** Climate change, Diurnal temperature range, Childhood asthma

## Abstract

**Background:**

Hot and cold temperatures have been associated with childhood asthma. However, the relationship between daily temperature variation and childhood asthma is not well understood. This study aimed to examine the relationship between diurnal temperature range (DTR) and childhood asthma.

**Methods:**

A Poisson generalized linear model combined with a distributed lag non-linear model was used to examine the relationship between DTR and emergency department admissions for childhood asthma in Brisbane, from January 1st 2003 to December 31st 2009.

**Results:**

There was a statistically significant relationship between DTR and childhood asthma. The DTR effect on childhood asthma increased above a DTR of 10°C. The effect of DTR on childhood asthma was the greatest for lag 0–9 days, with a 31% (95% confidence interval: 11% – 58%) increase of emergency department admissions per 5°C increment of DTR. Male children and children aged 5–9 years appeared to be more vulnerable to the DTR effect than others.

**Conclusions:**

Large DTR may trigger childhood asthma. Future measures to control and prevent childhood asthma should include taking temperature variability into account. More protective measures should be taken after a day of DTR above10°C.

## Background

Childhood asthma is a major global health issue, affecting more than 300 million people worldwide [1] and is regarded as a national health priority in several countries [2,3]. It was the most commonly reported long-term condition among 0–14 years Australian children in 2007–2008 [4]. Although the underlying causes of childhood asthma are not well understood, previous studies have documented that a variety of factors, including viral infections [5], air pollution [6] and aero-allergen exposure [7], contribute to the onset of asthma attacks.

Ambient temperature is also associated with childhood asthma [5,8]. However, there has been limited research on the relationship between daily temperature variation and childhood asthma. Diurnal temperature range (DTR), the temperature variation within one day, has been associated with cardiovascular and respiratory diseases [9-12]. For example, Song et al. postulated that large DTR may be a source of additional environmental stress in chronic obstructive pulmonary disease, and it has been found that stress on the respiratory system increases during periods of extreme temperature change [10]. Thus, it is probable that large DTR could also pose a threat to childhood asthma. Nonetheless, to date, few data are available on this topic.

This study aimed to examine three key research questions: 1) What is the relationship between DTR and childhood asthma? 2) Which children subgroups are most vulnerable to DTR effect? 3) Is there a delayed effect of DTR on childhood asthma?

## Methods

### Data collection

Emergency department admission (EDA) data during January 1st 2003 – December 31st 2009 were obtained from Queensland Health and were classified according to the International Classification of Disease, 10th version (ICD–code 10). Ethical approval was obtained prior to the data being collected. EDAs with the principle cause coded as asthma (ICD–10 codes: J45) among children aged 0–14 years were included in the data analysis. Daily data on maximum and minimum temperatures and relative humidity in Brisbane for the same period were retrieved from the Australian Bureau of Meteorology. DTR was calculated as the daily maximum temperature minus the daily minimum temperature. Data on daily average particular matter ≤ 10 μm (PM_10_) (μg/m^3^), daily average nitrogen dioxide (NO_2_) (μg/m^3^) and daily average ozone (O_3_) (ppb) were obtained from the Queensland Department of Environment and Heritage Protection.

### Statistical analyses

A Poisson generalized linear regression model combined with a distributed lag non-linear model (DLNM) was used to quantify the effect of DTR on childhood asthma. NO_2_, PM_10_, O_3_, mean temperature and relative humidity were controlled in the model by using a natural cubic spline with three degrees of freedom (*df*). Seasonal patterns and long-term trends were controlled by using a natural cubic spline with seven *df* per year of data. Day of week was controlled as a categorical variable, and influenza epidemics were also controlled. In all cases, the Akaike Information Criterion (AIC) and an analysis of residuals were used to evaluate the choice of *df*.

Previous studies have revealed that there may be a lagged effect of DTR on human health [10,13]. Further, the relationship between DTR and respiratory diseases has been shown to be non-linear [11]. Therefore, we used a distributed lag non-linear model to incorporate both the lagged and non-linear DTR effects [14]. Specifically, DTR and lag were incorporated using a “natural cubic spline–natural cubic spline” approach. The model included lags up to 11 days, which corresponded to a relatively low AIC value, and also allowed to capture any harvesting effects due to large DTR days. We also found the effect of DTR on childhood asthma was negligible for lags above 11 days, so we calculated the relative risk of DTR with lags up to 11 days. Effects of DTR on childhood asthma admissions were estimated and presented as overall relative risks incorporating all lags, stated for a 5°C increase in DTR above 10°C. This value was selected by examining the DTR–asthma relationship graphically.

All data analysis was conducted using the R statistical environment (version 2.15) with the “dlnm” package used to fit the regression model [15]. A sensitivity analysis was performed by varying the *df* for time, temperature and humidity.

## Results

There were 13324 EDAs for asthma among children aged 0–14 years in Brisbane during 2003–2009. Table 1 shows the summary statistics of weather variables, air pollutants and age- and gender-specific asthma admissions. The average values of daily EDAs for asthma among children aged 0–4, 5–9, and 10–14 years were 3.4 (standard deviation (SD):2.4), 1.3 (SD:1.4) and 0.5 (SD:0.8), respectively. The average daily EDAs in male and female children were 3.2 (SD:2.4) and 2.0 (SD:1.7). The average values of daily mean, maximum, minimum temperatures and DTR were 20.6°C, 25.6°C, 15.5°C, and 10.0°C, respectively. Every year, there were 173 days with DTR over 10.0°C, and there were 32 days with DTR over 15.0°C, from 2003 to 2009. The average relative humidity was 57.3%, and the mean values of O_3_, PM_10_ and NO_2_ were 12.6 ppb, 16.0 μg/m^3^ and 7.0 μg/m^3^, respectively.

**Table 1:** Summary statistics for climate variables, air pollutants, and pediatric asthma in Brisbane, Australia, during January1st 2003 to December 31st 2009

**Variables**	**Median**	**Range**	**Percentile**	
			**25**	**75**
Mean temperature (°C)	20.9	9.0–34.2	17.3	23.8
Maximum temperature (°C)	25.8	13.8–40.2	23.0	28.1
Minimum temperature (°C)	16.1	−0.1–28.1	11.7	19.8
Diurnal temperature range (°C)	9.8	0.6–21.6	7.5	12.2
Relative humidity (%)	58.0	5.0–98.0	49.0	67.0
O_3_ (ppb)	12.3	1.7–31.6	9.9	14.9
PM_10_ (μg/m^3^)	14.5	4.4–355.2	11.7	17.9
NO_2_ (μg/m^3^)	6.0	0–25.3	3.8	9.7
EDAs for childhood asthma	5	0–23	3	7
0–4 asthma	3	0–15	2	5
5–9 asthma	1	0–9	0	2
10–14 asthma	0	0–6	0	1
Male asthma	3	0–15	1	5
Female asthma	2	0–11	1	3

Table 2 depicts the spearman correlations between weather variables and air pollutants. DTR was negatively correlated with mean temperature (r= −0.55) and humidity (r= −0.65). Further, there were statistically positive correlations between DTR and O_3_ (r= 0.15), PM_10_ (r= 0.06), and NO_2_ (r= 0.51).

**Table 2:** Spearman’s correlation coefficients between daily weather conditions and air pollutants in Brisbane, Australia, from January 1st 2003– December 31st 2009

	**Mean temperature**	**DTR**	**Relative humidity**	**PM**_**10**_	**O**_**3**_
DTR	−0.55**				
Relative humidity	0.39**	−0.65**			
PM_10_	0.08**	0.06**	−0.10**		
O_3_	0.04*	0.15**	−0.09**	0.16**	
NO_2_	−0.67**	0.51**	−0.33**	0.01	−0.22**

*P<0.05; ** P<0.01.

**Figure 1:**
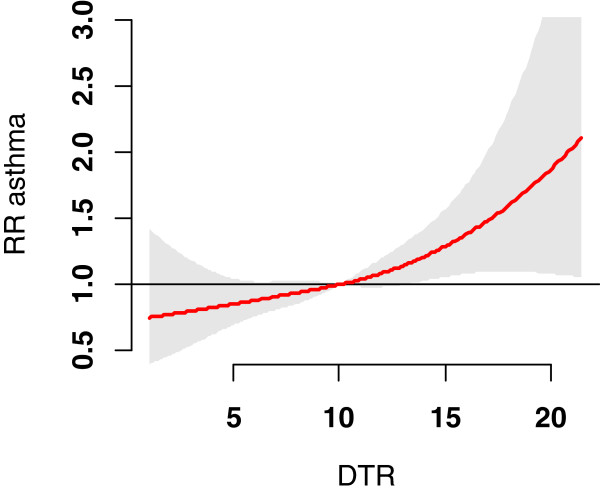
**The overall effects of diurnal temperature range on childhood asthma.** No reference was chosen, and the figure is for lag 0–11. NO_2_, PM_10_, O_3_, mean temperature and relative humidity were controlled.

Figure 1 shows the overall effects of DTR on childhood asthma up to 11 days. This figure does not suggest a threshold level of DTR associated with childhood asthma, but it does suggest that the DTR effect increased significantly above a DTR of 10°C (the average value across the study period).

Table 3 depicts the cumulative effects of DTR on childhood asthma. It indicates that DTR was more likely to affect asthma among male children and children aged 5–9 years. A 5°C increase (for lag 0–9) of DTR was found to be associated with a 31% increase in total, 81% increase in 5–9 year olds and 42% increase in male asthma admissions.

**Table 3:** The cumulative effects of diurnal temperature range on pediatric asthma, with diurnal temperature range of 15°C versus 10°C

	**Relative risk (95% CI)**							
	**Lag 0**	**Lag 0–1**	**Lag 0–2**	**Lag 0–3**	**Lag 0–8**	**Lag 0–9**	**Lag 0–10**	**Lag 0–11**
**Total children**	1.01 (0.97,1.06)	1.03 (0.97,1.13)	1.06 (0.98,1.19)	1.13 (1.02,1.25)*	1.31 (1.11,1.57)*	1.31 (1.11,1.58)*	1.31 (1.10,1.58)*	1.29 (1.06,1.57)*
**0-4 children**	0.99 (0.94,1.05)	0.98 (0.90,1.06)	1.00 (0.88,1.13)	1.02 (0.90,1.16)	1.17 (0.94,1.46)	1.18 (0.92,1.48)	1.15 (0.91,1.45)	1.12 (0.88,1.43)
**5-9 children**	1.09 (1.01,1.20)*	1.20 (1.05,1.37)*	1.29 (1.09,1.53)*	1.38 (1.13,1.69)*	1.75 (1.25,2.45)*	1.81 (1.26,2.58)*	1.86 (1.25,2.69)*	1.91 (1.26,2.83)*
**10-14 children**	1.01 (0.89,1.18)	1.07 (0.86,1.33)	1.15 (0.87,1.51)	1.24 (0.90,1.70)	1.54 (0.91,2.62)	1.49 (0.85,2.60)	1.38 (0.76,2.49)	1.23 (0.66,2.30)
**Male children**	1.02 (0.97,1.08)	1.06 (0.97,1.16)	1.12 (0.99,1.24)	1.18 (1.04,1.34)*	1.43 (1.15,1.77)*	1.42 (1.12,1.75)*	1.41 (1.08,1.74)*	1.35 (1.05,1.74)*
**Female children**	0.99 (0.92,1.03)	1.02 (0.91,1.13)	1.03 (0.90,1.19)	1.05 (0.90,1.24)	1.15 (0.88,1.52)	1.17 (0.88,1.56)	1.18 (0.87,1.59)	1.18 (0.86,1.63)

*P-value<0.05.

To conduct the sensitivity analysis, we changed the *df* (8–15 per year) for time to control for season. We also varied the *df* (4–7) for temperature and humidity. The results changed little (results not shown).

## Discussion

This study yielded several interesting findings. There was a statistically significant relationship between DTR and childhood asthma. The DTR effect increased significantly above a DTR of 10°C. The DTR effect was greatest for lag 0–9 days, and for children aged 5–9 years, with effects increasing for further lags. Male children and children aged 5–9 years appeared to be more vulnerable to the DTR effect than other children.

The results of this study support that there is a significant relationship between DTR and childhood asthma. In line with previous studies, Mireku et al. found that between-day changes of temperature were associated with emergency department visits for childhood asthma [16]. Even though the exact mechanism by which exposure to large DTR values increases asthma risk in children still remains to be explored, there are several plausible explanations for their relationship. Bull discussed that weather change might affect humoral or cellular immunity [17]. Children’s immune systems are relatively under-developed [18], and have less self-care ability [19], meaning that they may not dress appropriately and thus may have more thermal stress. When facing a large temperature change within one day, their thermoregulation cannot offset the pressure caused by the temperature change, and thus disease may occur. Togias et al. reported that sudden changes in the temperature of inhaled air was associated with the release of inflammatory mediators by mast cells [20]. Graudenz et al. argued that sudden temperature changes caused more inflammatory nasal responses [21], which may also explain some of the mechanisms associated with the DTR effect on asthma.

This study shows that there was no threshold level of DTR associated with childhood asthma, which corresponds to the results in other DTR studies [9,13]. Every year between 2003 and 2009, children in Brisbane were exposed to the risk of relatively large DTR (>10°C) for over 170 days, and to the risk of large DTR (>15°C) for over 30 days. The risk of childhood asthma increased significantly above the DTR of 10°C, suggesting that children, especially those with asthma, should be made aware of the possible threat posed by large DTR. This study indicates that large DTR values may trigger potential symptoms of asthma among children.

This study found that male children and children aged 5–9 years were more likely to suffer from asthma during days with large DTR. According to a report published by Australian Institute of Health and Welfare, males aged 5–9 represented the highest asthma prevalence group among all ages in Australia, accounting for approximately 15% of the total asthma patients [4]. It has been observed that boys are more prone to asthma than girls up to teenage years, which can be explained by differential growth rates of lung/airway size, along with immunological differences [22-24]. The reasons why children aged 5–9 are more vulnerable to asthma are unclear. This finding may encourage future studies concentrating on the physiological explanations of this vulnerability. A better understanding of vulnerable subgroups can give important clues to the children caregivers for better early protection.

This study is, to our knowledge, the first study which has found the statistically significant relation between childhood asthma and daily temperature variation in a subtropical city. Our results suggest that DTR may trigger childhood asthma, and future research needs to elucidate the causal pathway of this process. Additionally, we used advanced methods to assess this association, which was able to incorporate not only non-linear effects of temperature and DTR but also lagged effects in the one model.

Several limitations of this study should be acknowledged. This is an ecological study, and therefore some bias due to exposure misclassification may be inevitable. Only one city was examined, which might limit the generalizability of our results. However, the findings of this research may encourage a large scale, multi-centre study in the future.

## Conclusions

This study demonstrates a significant relationship between DTR and childhood asthma. Large DTR may increase the incidence of childhood asthma among children. As climate change continues, DTR is likely to increase in some regions of Australia, and it will be necessary to develop effective strategies to protect children from being harmed by climate impacts.

## Abbreviations

AIC: Akaike’s Information Criterion; DLNM: Distributed lag non-linear model; DTR: Diurnal temperature range; EDA: Emergency department admission; ICD10: International classification of disease, tenth revision; NO_2_: Nitrogen dioxide; PM_10_: Particulate matter ≤10 μm; SD: Standard deviation.

## Competing interests

The authors declare that they have no competing interests.

## Authors’ contributions

ZX and ST contributed to the design. ZX led in writing the paper. CH, HS, LRT and ZQ contributed to manuscript revision. All authors read and approved the final manuscript.
